# Identification of valid reference genes for microRNA expression studies in a hepatitis B virus replicating liver cell line

**DOI:** 10.1186/s13104-016-1848-2

**Published:** 2016-01-22

**Authors:** Kari Stougaard Jacobsen, Kirstine Overgaard Nielsen, Thilde Nordmann Winther, Dieter Glebe, Flemming Pociot, Birthe Hogh

**Affiliations:** Department of Paediatrics, Hvidovre Hospital, University of Copenhagen, Copenhagen, Denmark; Department of Paediatrics and Center for Non-Coding RNA in Technology and Health, Herlev Hospital, University of Copenhagen, Copenhagen, Denmark; Institute of Medical Virology, National Reference Center for Hepatitis B and D Viruses, German Center for Infection Research, Biomedical Research Center Seltersberg, Justus-Liebig University Giessen, Giessen, Germany

**Keywords:** Reference genes, Normalisation, microRNA, Hepatitis B virus, RT-qPCR

## Abstract

**Background:**

MicroRNAs are regulatory molecules and suggested as non-invasive biomarkers for molecular diagnostics and prognostics. Altered expression levels of specific microRNAs are associated with hepatitis B virus infection and hepatocellular carcinoma. We previously identified differentially expressed microRNAs with liver-specific target genes in plasma from children with chronic hepatitis B. To further understand the biological role of these microRNAs in the pathogenesis of chronic hepatitis B, we have used the human liver cell line HepG2, with and without HBV replication, after transfection of hepatitis B virus expression vectors. RT-qPCR is the preferred method for microRNA studies, and a careful normalisation strategy, verifying the optimal set of reference genes, is decisive for correctly evaluating microRNA expression levels. The aim of this study was to provide valid reference genes for the human HCC-derived cell line HepG2.

**Results:**

A panel of 739 microRNAs was screened to identify the most stably expressed microRNAs, followed by a PubMed search identifying microRNAs previously used as reference genes. Sixteen candidate reference genes were validated by RT-qPCR. Reference gene stabilities were calculated first by standard deviations of ΔCt values and then by geNorm and NormFinder analyses, taking into account the amplification efficiency of each microRNA primer set. The optimal set of reference genes was verified by a target analysis using RT-qPCR on miR-215-5p.

**Conclusion:**

We identified miR-24-3p, miR-151a-5p, and miR-425-5p as the most valid combination of reference genes for microRNA RT-qPCR studies in our hepatitis B virus replicating HepG2 cell model.

**Electronic supplementary material:**

The online version of this article (doi:10.1186/s13104-016-1848-2) contains supplementary material, which is available to authorized users.

## Background

MicroRNAs are suggested as non-invasive biomarkers for molecular diagnostics and prognostics, and some microRNAs show promising results as therapeutic targets in human trials [1–3]. A single microRNA can have multiple downstream targets and affect a number of different networks and pathways [4, 5]. Even small changes in microRNA expression may therefore have implications for gene regulation in various physiological and pathophysiological states [1, 6].

Hepatitis B virus (HBV) infection is a world-wide problem and approximately 240 million people live with a chronic HBV infection. HBV is one of the leading causes of hepatocellular carcinoma (HCC) and is responsible for the death of around 780,000 people each year [7].

MicroRNAs are thought to play an important role in early HBV infection, chronic HBV infection, and HBV-related cirrhosis and HCC [8–10]. Chronic hepatitis B (CHB) in children is associated with a high risk of developing HCC over time [11, 12]. Current knowledge on the pathogenesis at the molecular level is, however, limited [13]. We previously identified a panel of differentially expressed microRNAs in plasma from children with CHB [14] and furthermore showed that a number of the identified microRNAs had liver-specific target genes [15]. Liver tissue samples are unavailable as the majority of children with CHB are asymptomatic. Instead we use an in vitro HBV-replicating liver cell model to investigate the biological role of microRNAs with a possible influence on the pathogenesis of CHB in children.

RT-qPCR is often the preferred method for microRNA expression studies because of its sensitivity, flexibility, wide dynamic range, accuracy, and high throughput [16, 17]. Correct normalisation of microRNA RT-qPCR data is important to avoid erroneous conclusions [18]. Currently, there is no standardised normalisation strategy for microRNA RT-qPCR expression studies [19]. The small nuclear RNA U6 is frequently used as a reference gene (RG) in such studies [20]. Another strategy that has achieved general acceptance in RT-qPCR studies is identification of valid RGs by computer software such as geNorm and NormFinder [21, 22].

The present study was conducted to identify suitable RGs for microRNA expression studies in a human HCC-derived cell line (HepG2 tet-on), with and without HBV replication, after transfection of HBV expression vectors. We performed a systematic evaluation and identified miR-24-3p, miR-151a-5p, and miR-425-5p as the most valid combination of RGs in our model system. Results were verified by assessment of miR-215-5p, which is known to have a role in the pathogenesis of HBV infection [23, 24].

## Results and discussion

### Identification of candidate reference genes

Experiments were performed in the human HCC-derived cell line HepG2 tet-on. HBV replication was introduced by a doxycycline-inducible vector containing the 3091 HBV genome [25].

We applied a human microRNA PCR panel (Exiqon, Vedbaek, Denmark) to identify a number of stably expressed microRNAs. The microRNA panel included 739 microRNAs, and according to the manufacturer it included microRNAs that are either (1) generally more highly expressed, (2) more likely to be differentially expressed in disease, or (3) more often cited in the literature. We investigated the overall expression at two different time points (48 and 72 h) in the control HepG2 tet-on cell line and the HBV-replicating cell line (DOXY) (See Additional file 1 Figure S1 for heatmap, and Additional file 2 Table S1 for raw C_T_ values). We identified the presence of 137 microRNAs, using an exclusion value of C_T_ ≥ 32. We then evaluated the overall stability of each microRNA by calculating the standard deviation (SD) for all samples. MicroRNAs with SD values below 0.2 (35 microRNAs in total) were further investigated in a PubMed search to identify the microRNAs previously used as RGs. On this basis, 13 microRNAs (miR-17-5p, -24-3p, -26b-5p, -93-5p, -103a-3p, -106a-5p, -130b-3p, -151a-5p, -191-5p, -221-3p, -425-5p, -940 and let-7d-5p) were selected as candidate RGs (Table 1). Three small nuclear non-coding RNAs (U6 snRNA, SNORD38B, and SNORD49A) that are often used as RGs in microRNA expression studies [20, 26–28] were included to test for the importance of structural similarities, when choosing appropriate RGs. In total, 16 non-coding RNAs were subjected to further investigation.

**Table 1:** Candidate reference genes for microRNA normalisation

microRNA	Sequence	PubMed ID
miR-17-5p	CAAAGUGCUUACAGUGCAGGUAG	18375788
miR-24-3p	UGGCUCAGUUCAGCAGGAACAG	22074795
miR-26b-5p	UUCAAGUAAUUCAGGAUAGGU	18718003
miR-93-5p	CAAAGUGCUGUUCGUGCAGGUAG	22134529 18375788
miR-103a-3p	AGCAGCAUUGUACAGGGCUAUGA	22134529 21519184 18375788
miR-106a-5p	AAAAGUGCUUACAGUGCAGGUAG	18375788 22788411
miR-130b-3p	CAGUGCAAUGAUGAAAGGGCAU	20890088
miR-151a-5p	UCGAGGAGCUCACAGUCUAGU	22745731
miR-191-5p	CAACGGAAUCCCAAAAGCAGCUG	22134529 21519184 18375788
miR-221-3p	AGCUACAUUGUCUGCUGGGUUUC	21567136
miR-425-5p	AAUGACACGAUCACUCCCGUUGA	20429937
miR-940	AAGGCAGGGCCCCCGCUCCCC	24488924
let-7d-5p	AGAGGUAGUAGGUUGCAUAGUU	24223986

### Individual RT-qPCR and amplification efficiency

The selected candidate RGs were analysed by RT-qPCR. MiR-103a-3p and miR-let-7d-5p were excluded from further analyses because of C_T_ values > 32. Mean C_T_ values ± SD are shown in Table 2.

**Table 2:** Mean Ct values for the 16 candidate reference genes

microRNA	Raw Ct value (mean)	SD
miR-221-3p	27.11	0.64
let-7d-5p	32.60	0.99
miR-106a-5p	24.32	0.35
miR-103a-3p	38.52	0.79
miR-93-5p	25.15	0.27
miR-17-5p	29.86	0.32
miR-26b-5p	28.54	0.41
miR-130b-3p	29.82	0.40
miR-24-3p	25.58	0.26
miR-425-5p	27.89	0.31
miR-191-5p	26.93	0.32
miR-151a-5p	27.19	0.24
miR-940	30.84	0.30
U6 snRNA	23.78	1.56
SNORD38B	23.36	0.38
SNORD49B	22.91	0.38

Reference genes with CT values > 32 were excluded from further analyses

We evaluated the primer amplification efficiencies of the remaining 14 non-coding RNAs. The optimal primer efficiency is 2.0 corresponding to a doubling of the target in each cycle. In the present setup we used Locked Nucleic Acid (LNA) primers [29] and obtained amplification efficiencies from 1.8 to 2.7 (efficiencies and R^2^ values are listed in Table 3). LNA primers have been shown to have low amplification efficiencies [30] but were used in this study to ensure high affinity, specificity, and stability with regards to the microRNAs of interest [17, 31]. Calculated primer efficiencies were used in both our geNorm and NormFinder analyses [32].

**Table 3:** PCR amplification efficiencies

microRNA	Standard curve		E = 10^−1/slope^	
	Slope	R^2^	Primer efficiency	Efficiency (%)
miR-130b-3p	−3.829	0.996	1.825	82
miR-93-5p	−3.805	0.999	1.832	83
miR-221-3p	−3.765	0.996	1.843	84
miR-106a-5p	−3.746	0.999	1.849	85
miR-425-5p	−3.745	0.989	1.849	85
miR-191-5p	−3.688	0.988	1.867	87
miR-24-3p	−3.577	0.994	1.904	90
miR-151a-5p	−3.576	0.997	1.904	90
miR-940	−3.316	0.993	2.003	100
SNORD38B	−3.231	0.996	2.040	104
miR-26b-5p	−2.975	0.948	2.168	117
miR-17-5p	−2.884	0.933	2.222	122
SNORD49A	−2.836	0.991	2.252	125
U6 snRNA	−2.267	0.791	2.761	176

### Stability analysis by ∆Ct, geNorm and NormFinder

**Fig. 1:**
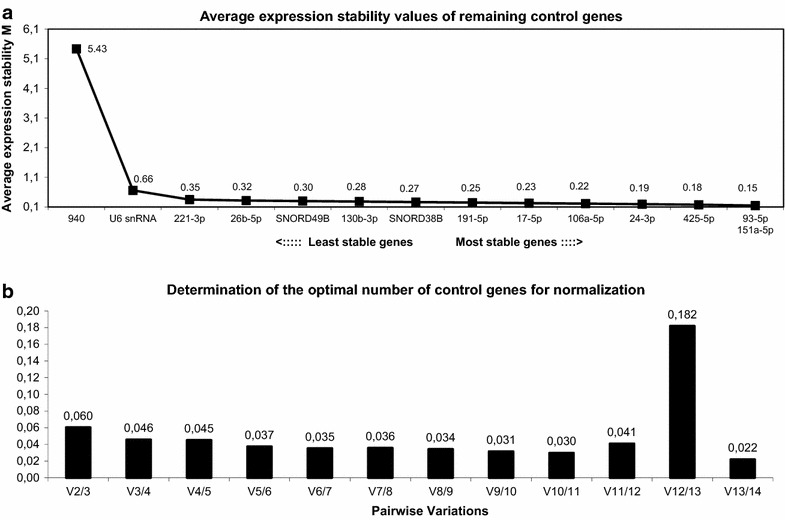
geNorm analysis of candidate reference genes. **a** Ranking of reference genes according to the average expression stability M. A stepwise exclusion strategy identified miR-93-5p and miR-151a-5p as the most stable reference gene pair. **b** Determination of the optimal number of reference genes for normalisation, concluding that top three most stable reference genes would suffice for correct normalisation

Overall expression stability of our 14 candidate RGs was evaluated by calculating the SD of the ∆Ct values from samples taken over time (24–96 h) in the cell lines: HepG2 tet-on, DOXY, and its negative control containing the non-replicating HBV genome. Top three were miR-151a-5p, miR-24-3p and miR-93-5p with SD values < 0.3. geNorm selects the optimal number of RGs based on the calculation of a stability value (M) and a pairwise variation value (V). In our analysis, miR-93-5p and miR-151-3p were identified as the most stable RG pair (M = 0.15) (Fig. 1a). A pairwise variation analysis showed that two RGs should suffice (V = 0.06), but adding a third gene (miR-425-5p) would improve normalisation (V = 0.05) (Fig. 1b). Three is also the lowest number of RGs recommended for use in microRNA expression studies [33]. One microRNA, miR-940, had an M-value above the exclusion limit of 1.5 (M = 5.43), making it unsuitable as a RG (Fig. 1a). NormFinder identifies the best normalisation gene from a group of candidates by calculating a stability value for each candidate. Our analysis revealed miR-93-5p as the best RG, with a stability value of 0.04. The best combination of genes was miR-130b-3p and miR-24-3p, lowering the stability value to 0.025 (Table 4).

**Table 4:** NormFinder results. Candidate reference genes ranked according to expression stability

Rank	Gene	Stability
1	miR-93-5p	0.040
2	miR-191-5p	0.076
3	miR-151a-5p	0.084
4	miR-425-5p	0.106
5	miR-24-3p	0.124
6	miR-130b-3p	0.134
7	miR-17-5p	0.141
8	SNORD38B	0.157
9	miR-940	0.167
10	SNORD49B	0.190
11	miR-106a-5p	0.213
12	miR-26b-5p	0.255
13	miR-221-3p	0.401
14	U6 snRNA	1.427
Best combination	miR-130b-3p/miR-24-3p	0.025

**Fig. 2:**
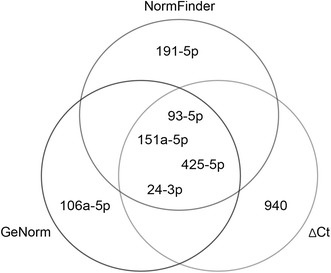
Venn diagram showing the top five candidates from the three different approaches (*∆Ct*, *geNorm* and *NormFinder*) and their overlap

A top-five comparison between the three approaches examined above found miR-151a-5p, miR-93-5p, miR-24-3p, and miR-425-5p to be the best RGs. According to geNorm three RGs are sufficient for optimal normalisation. Taking the amplification efficiencies into account (Table 3), the best combination to use in the present model system is miR-151a-5p, miR-425-5p, and miR-24-3p (Fig. 2).

It is worth noting that U6, which is often used as a RG in microRNA RT-qPCR studies, had the most deviated amplification efficiency (E = 2.761) and ranked last or second to last in the three analyses.

### Validation of the cell model system

We make use of the human liver cell model HepG2 tet-on for our in vitro studies due to lack of primary tissue from asymptomatic children with CHB. We introduced a doxycycline-inducible vector containing the HBV genome and the transactivator controlling mature virus production. Two genes work independently of this transactivator: The S gene, coding for the three surface proteins present in the viral envelope, and the X gene found to have oncogenic properties [34]. These proteins are continuously expressed independently of doxycycline-induction.

To evaluate our model system we investigated the expression of the most abundant liver specific microRNA, miR-122. miR-122 has been found to inhibit the expression and replication of HBV, and it is found down-regulated upon HBV infection, possibly facilitating viral replication and persistence [35–37]. We found the expression of miR-122-5p significantly decreased upon the insertion of the HBV plasmid independent of HBV replication [C_T_ = 24 in the HepG2 tet-on cell line and C_T_ = 34 in the cell lines containing the HBV genome with or without induction (p < 0.0001)]. This is in accordance with results from previous studies identifying the HBx protein as a negative regulator of miR-122 expression [37, 38], and the constitutive expression of the HBx protein in the transfected cell lines. This result supports the usage of this cell model system to investigate the biological role of microRNAs in HBV infection.

### Validation of reference genes

To assess the reliability of our selected RGs, we performed a target analysis on miR-215-5p, previously found to be up-regulated in CHB in children and adults as well as in HBV-related HCC [14, 23, 24, 39]. We studied the expression of miR-215-5p in HepG2 cells with and without HBV replication by RT-qPCR after 48 h of doxycycline treatment, and we normalised using miR-24-3p, miR-151a-5p, and miR-425-5p. Fold change was calculated using the 2^−∆∆Ct^ method [40]. We found a significant up-regulation of miR-215-5p in the DOXY cell line compared to the control HepG2 tet-on cell line (0.2 fold, p = 0.04). We also normalised against the commonly used RG U6 and found miR-215-5p to be up-regulated by 11.2 fold (p = 0.01). The raw C_T_ values showed approximately 1 cycle between HepG2 tet-on and DOXY. U6 is thus not a robust RG in this model system. Several authors have expressed concern about the use of this specific spliceosomal RNA as RG in microRNA studies because of its variability in structure and abundance as well as its often high degree of variance across samples [19, 20, 41, 42]. Our study demonstrates the importance of correct normalisation in microRNA expression studies, where even small changes in expression can have a vast downstream effect [5]. Comparison across studies requires validated RGs to avoid analytical errors and contradictory results [16, 19].

## Conclusion

We evaluated 16 small non-coding RNAs as possible RGs in a HBV-replicating liver cell line using ∆C_T_ and the two software algorithms geNorm and NormFinder. We identified miR-24-3p, miR-151a-5p, and miR-425-5p as the most valid combination of RGs to use for microRNA studies in this HBV-replicating HepG2 cell system and confirmed their validity with miR-215-5p. This result establishes a solid foundation for further studies in this cell culture model.

## Methods

### Cell lines and cell culture

The HepG2 tet-on Advanced Cell Line was purchased from Clontech (California, USA), cultured in DMEM media with 2 % fetal calf serum (FCS), 1 % penicillin/streptomycin, and G418 (100 µg/ml) at 37° C with 5 % CO_2_. To produce an HBV-replicating cell line, we introduced a doxycycline-inducible vector that contained the transactivator and an HBV genome encoding for HBV genotype D (ayw3 serotype) [25, 43]. The transactivator controls the synthesis of the HBV pregenomic mRNA and thus expression of the Polymerase gene and the PreCore/Core gene, while the Surface proteins gene and the X gene are transcribed continuously using their individual promoters. HBV replication was activated by applying doxycycline (1 µg/ml) to the media.

### Verification of viral replication

Media and cells were sampled at 7 time points at 12 h intervals (24–96 h). Media was changed following the 48 h sampling to ensure optimal growth conditions. All cell lines were tested for HBV replication prior to further analyses. HBsAg was measured from the media using an in-house sandwich ELISA [C20/2, sample, Anti-HBs-Biotin, Streptavidin-Peroxidase and substrate (OPD)] as previously described [44]. The cell lines transfected with the vector containing the HBV genome with and without doxycycline showed similar amounts of HBsAg in their media, corresponding to the design explained in the section above. HBV DNA was only measured in media from the HBV-replicating cell line (data not shown).

### RNA extraction and cDNA synthesis

Total RNA was extracted from cell lines using the miRNeasy Mini Kit (Qiagen, Hilden, Germany) in accordance with the manufacturer’s instructions, with minor modifications (200 µL chloroform was added, and after samples were applied to RNeasy Mini spin columns all centrifugation steps were performed at room temperature (RT) at 13,000×*g*). Elution was performed once with 50 µL RNase-free water. RNA concentrations were determined and evaluated using the NanoDrop 2000c spectrophometer (Thermo Scientific Waltham, Massachusetts, USA), measuring the absorbance at 260 nm as well as the absorbance ratio 260/280 nm to check for proper RNA quality. RNA samples were stored at −80° C.

cDNA synthesis was performed using the Universal cDNA Synthesis Kit II (Exiqon, Vedbaek, Denmark) in accordance with the manufacturer’s instructions and conducted on a GeneAmp PCR System 9700 (Applied Biosystems, Carlsbad, California, USA). 10 ng RNA was used for each cDNA synthesis, and blank samples with H_2_O as template were included. cDNA was stored at −20° C until further use.

### Quantitative real-time PCR

#### microRNA PCR panels

microRNA expressions were analysed in the cell lines using human microRNA PCR Panels (Human Panel I and II V2.M) and miRCURY LNA™ Universal RT microRNA PCR system (Exiqon, Vedbaek, Denmark) as described previously [14]. To further narrow down the putative RG candidates, a PubMed search was performed on each candidate to identify the microRNAs that have previously been used as RGs in at least one publication.

#### Individual RT-qPCR

Quantification of microRNA expression levels were performed by RT-qPCR using the miRCURY LNA™ Universal RT microRNA PCR system and specific microRNA LNA™ PCR primer sets (Exiqon, Vedbaek, Denmark). Specific microRNA target sequences are shown in Table 1. cDNA was diluted 80× and run in accordance with Exiqon’s instructions manual on a CFX384 Real-Time thermal cycler (Biorad, Hercules, California, USA). Each sample was run in duplicate, and negative controls with no-template and blanks as well as a spike-in, UniSp6, were assayed in each run.

#### Amplification efficiency

Amplification efficiency for each microRNA LNA™ PCR primer set was determined by standard curve analysis. Dilution series were made from a pool of cDNA including samples from all cell lines and all time points. Each sample was run in duplicate, and blank samples were included as negative controls. A C_T_ cut-off value was set to 36 for all primer efficiency analyses. Standard curve analyses were performed in duplicate, and average amplification efficiencies were used for further analyses.

#### Analysis of RT-qPCR data

Expression stability of the testet RGs was analysed using two widely applied algorithms: geNorm [21] and NormFinder [22]. The geNorm algorithm calculates an M-value that represents a gene’s variation compared to other candidate genes. A stepwise exclusion strategy identifies the two most stable genes. A candidate RG with an M-value above 1.5 is not considered a suitable RG. geNorm also calculates a V-value that determines the optimal number of RGs for normalisation. Starting with the two most stably expressed genes, a third, fourth, fifth, etc. is added, illustrating the levels of variation in average reference gene stability. A V-score below 0.15 is adequate for normalisation. The NormFinder algorithm calculates a stability value. The gene with the lowest stability value is determined to be the most stable RG. NormFinder provides both the best stable RG as well as the best stable pair of RGs. This is done by taking the different subgroups into account, estimating the inter-group and intra-group variations across the samples, identifying potential co-regulation between the candidates in the different subgroups.

### Statistics

All data is represented as raw data or as mean ± SD. Statistical analyses were performed in GraphPad Prism 6 using the unpaired Student’s t test (two-tailed). p-values < 0.05 were considered significant.

## Additional files

10.1186/s13104-016-1848-2 Heatmap of microRNA screen performed on the raw Ct values.
10.1186/s13104-016-1848-2 Data from the human microRNA screen. Raw Ct values. Table S2. Target gene analysis for miR-24-3p and miR-151a-5p. Targets are predicted by TargetScan, miRanda and picTar with a CLIP-Seq overlap. Table S3. Target gene analysis for miR-425-5p. No CLiP-Seq data is available for this microRNA. Targets are therefore only predicted using TargetScan and only targets with evolutionary conserved target sites are listed.
