# Supplementary figures and images for: Identification of valid reference genes for microRNA expression studies in a hepatitis B virus replicating liver cell line
Source: BMC Res Notes. 2016 Jan 22;9:38. doi: 10.1186/s13104-016-1848-2 (PMC4724106; doi:10.1186/s13104-016-1848-2)

# Color Key

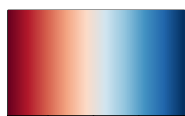

25 35

Ct Value

- HepG2-tet.48h
- HepG2-tet.72h
- DOXY.48h
- DOXY.72h

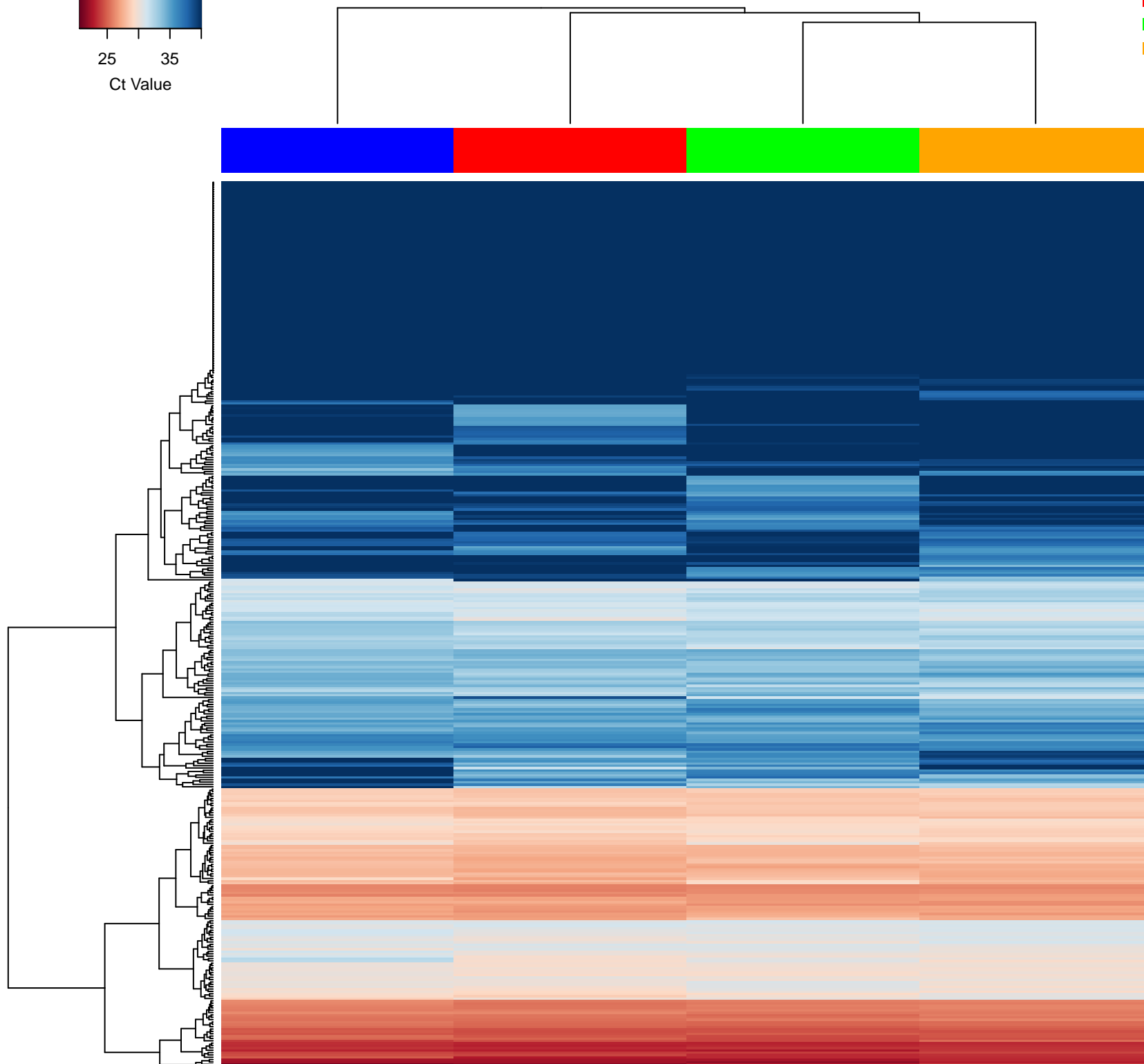

Supplement: Supplementary file 1 — 10.1186/s13104-016-1848-2 Heatmap of microRNA screen performed on the raw Ct values. [file 13104_2016_1848_MOESM1_ESM.pdf]
