# Supplementary material for: Identification of valid reference genes for microRNA expression studies in a hepatitis B virus replicating liver cell line
Source: BMC Res Notes. 2016 Jan 22;9:38. doi: 10.1186/s13104-016-1848-2 (PMC4724106; doi:10.1186/s13104-016-1848-2)
Supplement: Supplementary file 2 — 10.1186/s13104-016-1848-2 Data from the human microRNA screen. Raw Ct values. Table S2. Target gene analysis for miR-24-3p and miR-151a-5p. Targets are predicted by TargetScan, miRanda and picTar with a CLIP-Seq overlap. Table S3. Target gene analysis for miR-425-5p. No CLiP-Seq data is available for this microRNA. Targets are therefore only predicted using TargetScan and only targets with evolutionary conserved target sites are listed. [file 13104_2016_1848_MOESM2_ESM.docx]

**Table S1: Data from the human microRNA screen. Raw Ct values^[[1]](#footnote-1)^**

|  | **Raw Ct values** | | | | | | | |
| --- | --- | --- | --- | --- | --- | --- | --- | --- |
|  | **HepG2 tet-on** | | | | **DOXY** | | | |
| **miRNA** | **48h** | | **72h** | | **48h** | | **72h** | |
| hsa-miR-379 | 40,00 | | 38,25 | | 40,00 | | 40,00 | |
| hsa-miR-217 | 40,00 | | 40,00 | | 40,00 | | 40,00 | |
| hsa-miR-337-5p | 40,00 | | 40,00 | | 40,00 | | 40,00 | |
| hsa-miR-328 | 31,66 | | 31,28 | | 31,00 | | 31,86 | |
| hsa-miR-374b-3p | 40,00 | | 40,00 | | 40,00 | | 40,00 | |
| hsa-miR-143 | 30,28 | | 29,39 | | 29,51 | | 29,69 | |
| hsa-miR-623 | 40,00 | | 40,00 | | 40,00 | | 40,00 | |
| 520c-3p | 40,00 | | 39,26 | | 40,00 | | 37,68 | |
| hsa-miR-557 | 40,00 | | 40,00 | | 40,00 | | 40,00 | |
| hsa-miR-218 | 35,34 | | 40,00 | | 36,47 | | 39,26 | |
| hsa-miR-136 | 35,78 | | 40,00 | | 40,00 | | 40,00 | |
| hsa-miR-127-5p | 40,00 | | 40,00 | | 40,00 | | 40,00 | |
| hsa-miR-140-5p | 27,81 | | 27,78 | | 27,67 | | 27,99 | |
| hsa-miR-31-3p | 40,00 | | 40,00 | | 40,00 | | 40,00 | |
| hsa-miR-20b-3p | 32,41 | | 31,28 | | 31,52 | | 31,22 | |
| hsa-miR-325 | 40,00 | | 40,00 | | 40,00 | | 40,00 | |
| hsa-miR-509-3-5p | 40,00 | | 40,00 | | 40,00 | | 40,00 | |
| hsa-miR-210 | 28,02 | | 27,55 | | 27,04 | | 27,43 | |
| hsa-miR-199b-5p | 31,05 | | 30,90 | | 31,50 | | 32,94 | |
| hsa-miR-194 | 25,31 | | 24,63 | | 24,31 | | 24,69 | |
| hsa-let-7g | 28,27 | | 27,71 | | 27,62 | | 28,09 | |
| hsa-miR-203 | 40,00 | | 40,00 | | 40,00 | | 40,00 | |
| hsa-miR-181a-3p | 40,00 | | 33,81 | | 36,96 | | 33,60 | |
| hsa-miR-934 | 35,67 | | 35,60 | | 37,38 | | 36,48 | |
| hsa-miR-551b | 34,88 | | 40,00 | | 40,00 | | 40,00 | |
| UniSp6 CP | 21,82 | | 20,66 | | 22,91 | | 20,80 | |
| hsa-miR-524-3p | 40,00 | | 40,00 | | 40,00 | | 40,00 | |
| hsa-miR-7 | 35,36 | | 34,04 | | 35,28 | | 33,67 | |
| hsa-miR-486-5p | 36,74 | | 35,91 | | 35,07 | | 35,48 | |
| hsa-miR-30c | 25,70 | | 25,27 | | 25,08 | | 25,26 | |
| hsa-miR-301b | 29,49 | | 29,14 | | 29,27 | | 30,48 | |
| hsa-miR-128 | 30,07 | | 29,65 | | 30,02 | | 29,91 | |
| hsa-miR-329 | 40,00 | | 40,00 | | 40,00 | | 36,28 | |
| hsa-miR-224 | 30,64 | | 30,82 | | 30,81 | | 31,01 | |
| hsa-miR-487b | 40,00 | | 40,00 | | 40,00 | | 39,28 | |
| hsa-miR-130a | 30,56 | | 31,16 | | 30,63 | | 31,11 | |
| hsa-miR-138 | 34,19 | | 33,15 | | 33,53 | | 33,81 | |
| hsa-miR-26a-2-3p | 35,64 | | 38,00 | | 36,88 | | 35,98 | |
| hsa-miR-378 | 28,12 | | 27,37 | | 28,22 | | 28,30 | |
| hsa-miR-381 | 40,00 | | 40,00 | | 40,00 | | 40,00 | |
| hsa-miR-671-5p | 34,55 | | 33,98 | | 33,16 | | 33,21 | |
| hsa-miR-521 | 40,00 | | 34,67 | | 40,00 | | 40,00 | |
| hsa-miR-221 | 26,39 | | 26,33 | | 26,25 | | 26,41 | |
| hsa-miR-142-5p | 36,52 | | 37,37 | | 36,11 | | 40,00 | |
| hsa-miR-132 | 31,56 | | 30,52 | | 32,00 | | 32,38 | |
| hsa-miR-424 | 40,00 | | 40,00 | | 37,25 | | 40,00 | |
| UniSp3 IPC | 19,40 | | 18,94 | | 19,25 | | 19,81 | |
| hsa-miR-374a | 29,42 | | 28,29 | | 29,53 | | 29,37 | |
| hsa-miR-532-5p | 31,25 | | 29,49 | | 29,81 | | 29,71 | |
| hsa-miR-99a | 36,83 | | 35,66 | | 35,12 | | 36,35 | |
| hsa-miR-92a-1-5p | 30,98 | | 30,26 | | 29,86 | | 31,28 | |
| hsa-miR-125b | 40,00 | | 37,75 | | 39,55 | | 36,31 | |
| hsa-miR-185 | 32,29 | | 29,69 | | 30,56 | | 30,24 | |
| hsa-miR-25 | 25,89 | | 25,54 | | 25,78 | | 26,07 | |
| hsa-miR-524-5p | 40,00 | | 40,00 | | 34,80 | | 40,00 | |
| hsa-miR-20a | 23,33 | | 22,97 | | 23,19 | | 23,41 | |
| hsa-miR-765 | 40,00 | | 35,17 | | 40,00 | | 40,00 | |
| hsa-miR-24 | 24,65 | | 24,42 | | 24,44 | | 24,81 | |
| hsa-miR-369-5p | 40,00 | | 40,00 | | 40,00 | | 40,00 | |
| hsa-miR-425 | 27,10 | | 26,68 | | 27,00 | | 27,14 | |
| hsa-miR-590-5p | 29,00 | | 28,78 | | 28,52 | | 28,67 | |
| hsa-miR-760 | 33,36 | | 32,81 | | 32,77 | | 32,40 | |
| hsa-miR-574-3p | 28,05 | | 27,50 | | 27,63 | | 27,72 | |
| hsa-miR-130b | 29,42 | | 29,19 | | 29,11 | | 29,49 | |
| hsa-let-7e | 34,59 | | 33,55 | | 34,63 | | 32,22 | |
| hsa-miR-133b | 35,06 | | 36,95 | | 40,00 | | 35,95 | |
| hsa-miR-542-5p | 40,00 | | 40,00 | | 35,30 | | 40,00 | |
| hsa-miR-23a | 26,14 | | 25,86 | | 26,26 | | 26,41 | |
| hsa-miR-193b | 25,31 | | 25,10 | | 24,77 | | 25,03 | |
| IPC | 19,49 | | 19,16 | | 19,26 | | 19,80 | |
| hsa-miR-518c-5p | 37,82 | | 40,00 | | 38,57 | | 38,13 | |
| hsa-miR-204 | 40,00 | | 40,00 | | 40,00 | | 40,00 | |
| hsa-miR-933 | 38,27 | | 40,00 | | 36,47 | | 37,33 | |
| SNORD49 A | 22,33 | | 22,27 | | 21,87 | | 22,89 | |
| hsa-miR-452 | 30,06 | | 30,44 | | 30,13 | | 29,86 | |
| hsa-miR-215 | 25,16 | | 25,07 | | 24,21 | | 24,41 | |
| hsa-miR-141 | 30,67 | | 30,03 | | 30,55 | | 31,11 | |
| hsa-miR-374b | 28,46 | | 28,60 | | 28,50 | | 28,27 | |
| hsa-miR-668 | 38,51 | | 40,00 | | 35,72 | | 40,00 | |
| hsa-miR-33a | 39,21 | | 39,14 | | 37,66 | | 38,90 | |
| hsa-miR-101 | 27,94 | | 27,69 | | 28,00 | | 27,23 | |
| hsa-miR-30c-2-3p | 37,73 | | 35,00 | | 36,62 | | 40,00 | |
| hsa-miR-331-3p | 30,20 | | 29,48 | | 29,89 | | 29,96 | |
| hsa-miR-340 | 38,41 | | 37,85 | | 39,59 | | 38,17 | |
| hsa-miR-196a | 40,00 | | 40,00 | | 36,02 | | 36,33 | |
| hsa-miR-888 | 40,00 | | 35,31 | | 35,32 | | 38,77 | |
| hsa-miR-330-3p | 33,04 | | 33,39 | | 34,46 | | 33,12 | |
| hsa-miR-570 | 32,33 | | 32,58 | | 33,48 | | 33,14 | |
| hsa-miR-518c | 35,58 | | 40,00 | | 40,00 | | 40,00 | |
| hsa-miR-200a | 28,71 | | 28,20 | | 28,71 | | 28,75 | |
| hsa-miR-188-5p | 33,30 | | 32,06 | | 32,41 | | 32,03 | |
| hsa-miR-26a | 27,76 | | 27,09 | | 27,70 | | 27,35 | |
| hsa-miR-99b | 26,76 | | 26,95 | | 26,34 | | 26,32 | |
| hsa-miR-431 | 32,84 | | 32,76 | | 32,22 | | 33,49 | |
| hsa-miR-23b | 24,80 | | 24,33 | | 24,47 | | 24,39 | |
| hsa-miR-367 | 40,00 | | 40,00 | | 40,00 | | 40,00 | |
| hsa-miR-505 | 30,77 | | 30,19 | | 30,10 | | 29,99 | |
| hsa-miR-18a | 25,46 | | 25,39 | | 25,82 | | 26,09 | |
| hsa-miR-92a | 24,59 | | 24,41 | | 24,86 | | 24,62 | |
| hsa-miR-500a | 33,53 | | 32,38 | | 33,06 | | 32,84 | |
| hsa-miR-887 | 37,37 | | 40,00 | | 36,42 | | 38,65 | |
| hsa-miR-491-3p | 40,00 | | 35,89 | | 36,52 | | 35,86 | |
| hsa-miR-423-3p | 26,07 | | 25,97 | | 26,11 | | 26,23 | |
| hsa-miR-126 | 33,36 | | 32,28 | | 33,41 | | 31,70 | |
| hsa-miR-622 | 34,24 | | 34,09 | | 33,38 | | 34,09 | |
| hsa-miR-376b | 40,00 | | 40,00 | | 36,07 | | 37,28 | |
| hsa-miR-302c | 40,00 | | 40,00 | | 40,00 | | 40,00 | |
| hsa-miR-185-3p | 40,00 | | 40,00 | | 39,84 | | 39,49 | |
| hsa-miR-339-5p | 28,92 | | 28,39 | | 28,28 | | 28,50 | |
| hsa-miR-873 | 40,00 | | 35,33 | | 38,89 | | 40,00 | |
| hsa-miR-323-3p | 40,00 | | 40,00 | | 34,59 | | 40,00 | |
| hsa-miR-181d | 35,26 | | 35,54 | | 34,30 | | 37,00 | |
| hsa-miR-125a-5p | 26,27 | | 26,14 | | 25,67 | | 25,74 | |
| hsa-miR-5 | 40,00 | | 37,89 | | 40,00 | | 40,00 | |
| hsa-miR-492 | 40,00 | | 40,00 | | 40,00 | | 40,00 | |
| hsa-miR-519d | 40,00 | | 40,00 | | 35,70 | | 40,00 | |
| UniSp3 IPC | 19,11 | | 18,88 | | 19,20 | | 19,66 | |
| hsa-miR-302d | 40,00 | | 40,00 | | 40,00 | | 40,00 | |
| hsa-miR-346 | 34,65 | | 40,00 | | 40,00 | | 40,00 | |
| hsa-miR-151-3p | 29,91 | | 29,49 | | 29,33 | | 29,31 | |
| hsa-miR-493 | 34,89 | | 34,65 | | 35,32 | | 39,49 | |
| hsa-miR-423-5p | 30,37 | | 29,63 | | 30,70 | | 29,54 | |
| hsa-miR-99a-3p | 40,00 | | 40,00 | | 40,00 | | 40,00 | |
| hsa-miR-10a | 40,00 | | 40,00 | | 40,00 | | 40,00 | |
| hsa-miR-202 | 36,07 | | 38,31 | | 40,00 | | 40,00 | |
| hsa-miR-10b | 40,00 | | 40,00 | | 39,46 | | 40,00 | |
| hsa-miR-503 | 40,00 | | 40,00 | | 40,00 | | 40,00 | |
| hsa-miR-890 | 40,00 | | 40,00 | | 40,00 | | 40,00 | |
| hsa-miR-30d | 30,79 | | 30,72 | | 30,77 | | 30,30 | |
| hsa-miR-514 | 36,46 | | 36,17 | | 35,28 | | 34,75 | |
| hsa-miR-16 | 40,00 | | 37,35 | | 40,00 | | 37,02 | |
| hsa-miR-150 | 37,01 | | 40,00 | | 40,00 | | 40,00 | |
| hsa-miR-654-5p | 40,00 | | 35,74 | | 35,75 | | 37,50 | |
| hsa-miR-545 | 34,83 | | 33,24 | | 36,28 | | 34,31 | |
| hsa-miR-29b-2-5p | 32,47 | | 32,36 | | 32,57 | | 33,11 | |
| hsa-miR-491-5p | 31,31 | | 30,55 | | 30,73 | | 31,08 | |
| hsa-miR-92b | 35,21 | | 34,75 | | 35,31 | | 34,06 | |
| hsa-miR-665 | 32,06 | | 31,54 | | 32,90 | | 32,29 | |
| hsa-miR-506 | 37,16 | | 38,49 | | 36,87 | | 39,90 | |
| hsa-miR-363 | 34,07 | | 33,08 | | 36,17 | | 39,48 | |
| hsa-miR-663 | 30,97 | | 31,77 | | 32,48 | | 33,06 | |
| hsa-miR-651 | 36,33 | | 35,85 | | 35,09 | | 35,34 | |
| 342-3p | 28,76 | | 28,22 | | 28,73 | | 28,65 | |
| hsa-miR-432 | 40,00 | | 40,00 | | 40,00 | | 40,00 | |
| hsa-miR-154-3p | 40,00 | | 40,00 | | 40,00 | | 40,00 | |
| hsa-miR-27a | 29,80 | | 28,39 | | 30,21 | | 28,43 | |
| hsa-miR-376c | 34,54 | | 35,44 | | 35,78 | | 36,57 | |
| hsa-miR-940 | 29,95 | | 30,07 | | 29,78 | | 30,31 | |
| hsa-miR-22-5p | 31,11 | | 30,97 | | 31,24 | | 32,18 | |
| hsa-miR-34c-5p | 39,30 | | 40,00 | | 35,53 | | 35,59 | |
| hsa-miR-885-5p | 34,53 | | 33,24 | | 34,46 | | 34,92 | |
| hsa-miR-320a | 25,78 | | 25,86 | | 26,04 | | 25,83 | |
| hsa-miR-18b | 25,57 | | 25,54 | | 25,43 | | 25,63 | |
| hsa-miR-187 | 35,85 | | 40,00 | | 34,82 | | 39,36 | |
| hsa-miR-516b | 40,00 | | 40,00 | | 40,00 | | 40,00 | |
| hsa-miR-302c-5p | 40,00 | | 40,00 | | 40,00 | | 40,00 | |
| hsa-miR-548b-3p | 31,39 | | 31,14 | | 31,38 | | 31,52 | |
| hsa-miR-186 | 28,81 | | 28,45 | | 29,24 | | 28,83 | |
| hsa-miR-199a-5p | 40,00 | | 40,00 | | 39,03 | | 40,00 | |
| hsa-miR-155 | 40,00 | | 40,00 | | 40,00 | | 40,00 | |
| hsa-miR-107 | 26,15 | | 25,48 | | 25,75 | | 25,81 | |
| hsa-miR-302b | 40,00 | | 40,00 | | 40,00 | | 40,00 | |
| hsa-miR-662 | 35,92 | | 39,28 | | 40,00 | | 39,11 | |
| hsa-miR-30a | 40,00 | | 37,98 | | 40,00 | | 40,00 | |
| hsa-miR-302d-5p | 38,41 | | 40,00 | | 40,00 | | 40,00 | |
| hsa-miR-484 | 28,06 | | 27,52 | | 27,30 | | 27,52 | |
| hsa-miR-337-5p | 31,69 | | 30,48 | | 31,34 | | 30,86 | |
| hsa-miR-494 | 40,00 | | 40,00 | | 40,00 | | 40,00 | |
| hsa-miR-371-3p | 40,00 | | 34,67 | | 34,22 | | 34,52 | |
| hsa-miR-103 | 23,59 | | 23,32 | | 23,57 | | 23,57 | |
| hsa-miR-144 | 40,00 | | 35,32 | | 40,00 | | 40,00 | |
| hsa-miR-184 | 40,00 | | 40,00 | | 40,00 | | 37,00 | |
| hsa-miR-631 | 40,00 | | 40,00 | | 40,00 | | 34,01 | |
| hsa-miR-519a | 37,11 | | 36,32 | | 40,00 | | 35,49 | |
| hsa-miR-211 | 40,00 | | 40,00 | | 40,00 | | 40,00 | |
| hsa-miR-802 | 40,00 | | 40,00 | | 40,00 | | 40,00 | |
| hsa-let-7f | 29,21 | | 29,01 | | 29,43 | | 28,81 | |
| hsa-miR-625-3p | 31,62 | | 31,83 | | 32,25 | | 32,87 | |
| hsa-miR-34a | 25,59 | | 25,22 | | 25,08 | | 25,30 | |
| hsa-miR-744 | 30,08 | | 30,12 | | 30,75 | | 29,98 | |
| hsa-miR-518e | 40,00 | | 40,00 | | 40,00 | | 40,00 | |
| hsa-miR-29b | 27,64 | | 27,22 | | 27,94 | | 28,20 | |
| hsa-miR-658 | 40,00 | | 40,00 | | 40,00 | | 40,00 | |
| hsa-miR-572 | 40,00 | | 40,00 | | 40,00 | | 40,00 | |
| hsa-let-7a | 37,04 | | 34,67 | | 37,72 | | 33,76 | |
| hsa-miR-30e | 33,37 | | 31,56 | | 32,33 | | 32,22 | |
| hsa-miR-433 | 40,00 | | 40,00 | | 40,00 | | 40,00 | |
| hsa-miR-660 | 31,89 | | 30,00 | | 31,54 | | 30,75 | |
| hsa-let-7c | 31,44 | | 30,05 | | 30,57 | | 30,29 | |
| hsa-miR-28-5p | 27,22 | | 26,65 | | 26,91 | | 27,02 | |
| hsa-miR-324-5p | 27,32 | | 27,10 | | 26,72 | | 26,89 | |
| hsa-miR-219-5p | 31,00 | | 30,74 | | 31,35 | | 31,17 | |
| hsa-miR-19b | 24,81 | | 22,76 | | 23,29 | | 23,37 | |
| hsa-miR-526b | 40,00 | | 40,00 | | 40,00 | | 40,00 | |
| hsa-miR-720 | 21,04 | | 21,51 | | 22,87 | | 23,92 | |
| hsa-miR-30b | 26,00 | | 25,59 | | 25,98 | | 26,06 | |
| hsa-miR-637 | 40,00 | | 40,00 | | 40,00 | | 40,00 | |
| hsa-miR-422a | 40,00 | | 40,00 | | 40,00 | | 40,00 | |
| hsa-miR-199a-3p | 32,04 | | 31,80 | | 32,60 | | 32,76 | |
| hsa-miR-335 | 32,84 | | 33,07 | | 32,15 | | 32,28 | |
| hsa-miR-134 | 38,44 | | 33,84 | | 34,63 | | 38,23 | |
| hsa-miR-21 | 21,07 | | 20,60 | | 21,14 | | 21,41 | |
| hsa-miR-129-3p | 40,00 | | 40,00 | | 40,00 | | 40,00 | |
| hsa-miR-26b | 28,92 | | 28,55 | | 28,93 | | 28,76 | |
| hsa-miR-214 | 40,00 | | 40,00 | | 39,71 | | 40,00 | |
| hsa-miR-32 | 27,93 | | 27,95 | | 27,60 | | 27,69 | |
| hsa-miR-324-3p | 29,09 | | 29,04 | | 28,27 | | 28,70 | |
| hsa-miR-488 | 40,00 | | 40,00 | | 40,00 | | 40,00 | |
| hsa-miR-371-5p | 40,00 | | 36,85 | | 33,36 | | 35,21 | |
| hsa-miR-455-5p | 27,25 | | 27,02 | | 26,70 | | 26,92 | |
| hsa-miR-891a | 40,00 | | 40,00 | | 40,00 | | 40,00 | |
| hsa-miR-549 | 37,45 | | 36,83 | | 39,66 | | 40,00 | |
| hsa-miR-205 | 40,00 | | 40,00 | | 40,00 | | 40,00 | |
| hsa-miR-518b | 40,00 | | 40,00 | | 40,00 | | 40,00 | |
| hsa-miR-361-5p | 40,00 | | 40,00 | | 40,00 | | 40,00 | |
| hsa-miR-454 | 40,00 | | 40,00 | | 40,00 | | 40,00 | |
| hsa-miR-15a | 27,24 | | 26,82 | | 27,40 | | 26,66 | |
| hsa-miR-191 | 26,08 | | 25,94 | | 26,16 | | 26,15 | |
| hsa-miR-608 | 40,00 | | 40,00 | | 40,00 | | 40,00 | |
| hsa-miR-576-5p | 33,95 | | 34,74 | | 34,00 | | 35,55 | |
| hsa-miR-497 | 33,04 | | 32,11 | | 30,83 | | 31,69 | |
| hsa-miR-19a | 30,37 | | 30,02 | | 30,98 | | 29,75 | |
| hsa-miR-187-5p | 40,00 | | 40,00 | | 40,00 | | 40,00 | |
| hsa-miR-620 | 40,00 | | 40,00 | | 40,00 | | 40,00 | |
| hsa-let-7i | 32,32 | | 31,18 | | 31,67 | | 31,38 | |
| hsa-miR-501-5p | 30,34 | | 29,88 | | 30,50 | | 30,82 | |
| hsa-miR-652 | 28,68 | | 27,87 | | 28,16 | | 28,39 | |
| hsa-miR-1979 | 22,49 | | 22,36 | | 22,42 | | 23,25 | |
| hsa-miR-30e-3p | 31,29 | | 30,52 | | 31,02 | | 30,72 | |
| hsa-miR-181c | 34,09 | | 35,17 | | 35,43 | | 35,61 | |
| hsa-miR-499-5p | 39,53 | | 38,76 | | 38,07 | | 40,00 | |
| hsa-miR-548c-3p | 40,00 | | 34,75 | | 40,00 | | 40,00 | |
| hsa-miR-152 | 30,23 | | 29,64 | | 29,47 | | 30,05 | |
| hsa-miR-93 | 24,16 | | 23,85 | | 23,87 | | 24,15 | |
| hsa-miR-490-3p | 33,70 | | 33,02 | | 33,27 | | 34,20 | |
| hsa-miR-29c | 28,31 | | 27,93 | | 28,46 | | 28,58 | |
| hsa-miR-372 | 34,92 | | 38,74 | | 31,43 | | 31,09 | |
| hsa-miR-133a | 40,00 | | 34,89 | | 40,00 | | 36,20 | |
| hsa-miR-124 | 36,12 | | 38,82 | | 38,74 | | 39,15 | |
| hsa-miR-190 | 33,11 | | 32,90 | | 32,44 | | 32,11 | |
| hsa-miR-302a | 40,00 | | 40,00 | | 40,00 | | 40,00 | |
| hsa-miR-595 | 36,10 | | 39,31 | | 35,34 | | 40,00 | |
| hsa-miR-602 | 35,09 | | 37,91 | | 38,10 | | 38,97 | |
| hsa-miR-223 | 40,00 | | 40,00 | | 36,74 | | 40,00 | |
| hsa-miR-627 | 38,24 | | 37,71 | | 39,82 | | 37,41 | |
| hsa-miR-34b | 40,00 | | 40,00 | | 40,00 | | 40,00 | |
| hsa-miR-410 | 40,00 | | 37,77 | | 38,11 | | 40,00 | |
| hsa-miR-17 | 29,29 | | 29,01 | | 29,38 | | 29,07 | |
| hsa-miR-376a | 35,16 | | 34,04 | | 34,97 | | 36,43 | |
| hsa-miR-877 | 31,04 | | 31,12 | | 31,01 | | 30,94 | |
| hsa-miR-512-5p | 40,00 | | 40,00 | | 40,00 | | 37,47 | |
| hsa-miR-449a | 40,00 | | 40,00 | | 40,00 | | 40,00 | |
| hsa-miR-498 | 40,00 | | 40,00 | | 40,00 | | 40,00 | |
| hsa-miR-148b | 27,74 | | 27,13 | | 27,57 | | 27,58 | |
| hsa-miR-127-3p | 40,00 | | 40,00 | | 40,00 | | 40,00 | |
| hsa-miR-598 | 40,00 | | 36,57 | | 40,00 | | 40,00 | |
| hsa-miR-96 | 27,92 | | 27,85 | | 27,68 | | 28,27 | |
| hsa-let-7d | 31,40 | | 31,69 | | 31,59 | | 31,44 | |
| hsa-miR-135b | 26,92 | | 26,36 | | 27,10 | | 27,17 | |
| hsa-miR-495 | 40,00 | | 37,59 | | 40,00 | | 37,19 | |
| hsa-miR-299-5p | 39,84 | | 34,87 | | 40,00 | | 40,00 | |
| hsa-miR-34c-3p | 34,81 | | 34,67 | | 34,49 | | 34,97 | |
| hsa-miR-596 | 40,00 | | 40,00 | | 40,00 | | 40,00 | |
| hsa-miR-126-5p | 40,00 | | 37,38 | | 35,09 | | 40,00 | |
| hsa-miR-145 | 29,26 | | 26,98 | | 28,32 | | 27,52 | |
| SNORD38 B | 23,09 | | 22,89 | | 22,52 | | 23,68 | |
| hsa-miR-516a-5p | 40,00 | | 40,00 | | 40,00 | | 40,00 | |
| hsa-miR-421 | 29,60 | | 29,01 | | 29,06 | | 29,21 | |
| hsa-miR-96-3p | 40,00 | | 40,00 | | 40,00 | | 40,00 | |
| hsa-miR-362-5p | 31,44 | | 31,19 | | 31,48 | | 31,30 | |
| hsa-miR-615-3p | 40,00 | | 40,00 | | 40,00 | | 39,30 | |
| hsa-miR-550a | 33,54 | | 34,19 | | 33,55 | | 34,77 | |
| hsa-miR-766 | 31,07 | | 30,43 | | 30,35 | | 30,77 | |
| hsa-miR-200b | 27,30 | | 26,92 | | 28,30 | | 27,33 | |
| hsa-miR-298 | 35,78 | | 40,00 | | 37,25 | | 40,00 | |
| hsa-miR-193a-5p | 32,94 | | 32,87 | | 33,82 | | 34,10 | |
| hsa-miR-449b | 40,00 | | 40,00 | | 40,00 | | 40,00 | |
| hsa-miR-520d-5p | 40,00 | | 40,00 | | 40,00 | | 40,00 | |
| hsa-miR-192 | 24,37 | | 24,11 | | 23,28 | | 23,55 | |
| hsa-miR-29a | 25,92 | | 26,00 | | 26,73 | | 26,86 | |
| hsa-miR-18a-3p | 28,76 | | 28,69 | | 29,23 | | 29,21 | |
| hsa-miR-383 | 40,00 | | 40,00 | | 40,00 | | 40,00 | |
| hsa-miR-9 | 40,00 | | 40,00 | | 37,74 | | 38,53 | |
| hsa-miR-202-5p | 40,00 | | 40,00 | | 40,00 | | 40,00 | |
| hsa-miR-363-5p | 40,00 | | 40,00 | | 40,00 | | 40,00 | |
| hsa-miR-147b | 40,00 | | 40,00 | | 38,73 | | 40,00 | |
| hsa-miR-197 | 28,67 | | 28,40 | | 28,30 | | 28,80 | |
| hsa-miR-597 | 40,00 | | 40,00 | | 40,00 | | 36,85 | |
| hsa-miR-326 | 33,02 | | 32,41 | | 31,49 | | 32,36 | |
| hsa-miR-15b | 32,14 | | 29,47 | | 30,74 | | 29,46 | |
| hsa-miR-105 | 39,61 | | 34,76 | | 40,00 | | 40,00 | |
| hsa-miR-196b | 34,21 | | 34,25 | | 32,04 | | 32,07 | |
| hsa-miR-296-5p | 40,00 | | 35,37 | | 40,00 | | 40,00 | |
| hsa-miR-20b | 40,00 | | 40,00 | | 40,00 | | 40,00 | |
| hsa-miR-147 | 40,00 | | 40,00 | | 40,00 | | 40,00 | |
| hsa-miR-198 | 40,00 | | 40,00 | | 40,00 | | 40,00 | |
| hsa-miR-375 | 35,51 | | 35,47 | | 34,24 | | 34,22 | |
| hsa-miR-517a | 40,00 | | 37,31 | | 40,00 | | 40,00 | |
| hsa-miR-361-3p | 34,18 | | 33,03 | | 33,57 | | 32,92 | |
| hsa-miR-21-3p | 28,86 | | 29,95 | | 30,24 | | 30,83 | |
| hsa-miR-220a | 40,00 | | 40,00 | | 40,00 | | 40,00 | |
| hsa-miR-518f | 35,23 | | 40,00 | | 40,00 | | 40,00 | |
| hsa-miR-222 | 27,76 | | 27,13 | | 28,20 | | 27,87 | |
| hsa-miR-617 | 40,00 | | 40,00 | | 40,00 | | 40,00 | |
| hsa-miR-154 | 40,00 | | 40,00 | | 40,00 | | 40,00 | |
| hsa-miR-708 | 31,33 | | 40,00 | | 40,00 | | 33,87 | |
| hsa-let-7b | 33,90 | | 34,39 | | 34,79 | | 34,03 | |
| hsa-miR-95 | 40,00 | | 40,00 | | 40,00 | | 40,00 | |
| hsa-miR-517c | 35,39 | | 35,97 | | 39,42 | | 40,00 | |
| hsa-miR-151-5p | 26,23 | | 25,75 | | 25,84 | | 26,00 | |
| hsa-miR-502-5p | 28,31 | | 27,75 | | 28,51 | | 28,72 | |
| hsa-miR-345 | 34,01 | | 33,94 | | 33,19 | | 34,13 | |
| hsa-miR-509-3p | 40,00 | | 31,72 | | 34,11 | | 40,00 | |
| U6 | 23,91 | | 24,54 | | 24,23 | | 25,31 | |
| hsa-miR-382 | 40,00 | | 34,92 | | 40,00 | | 40,00 | |
| hsa-miR-373 | 38,59 | | 36,60 | | 32,50 | | 32,31 | |
| hsa-miR-200c | 30,02 | | 29,86 | | 29,78 | | 29,64 | |
| hsa-miR-9-3p | 40,00 | | 40,00 | | 40,00 | | 40,00 | |
| hsa-miR-181b | 29,61 | | 29,51 | | 30,54 | | 30,07 | |
| hsa-miR-628-3p | 33,80 | | 34,27 | | 34,36 | | 34,01 | |
| hsa-miR-195 | 37,44 | | 36,50 | | 38,89 | | 36,09 | |
| hsa-miR-183 | 30,60 | | 31,12 | | 31,05 | | 30,49 | |
| hsa-miR-135a | 29,88 | | 29,23 | | 30,18 | | 29,33 | |
| hsa-miR-30b-3p | 38,11 | | 38,28 | | 39,28 | | 37,75 | |
| hsa-miR-146b-5p | 31,32 | | 30,95 | | 31,99 | | 30,74 | |
| hsa-miR-301a | 33,09 | | 32,58 | | 34,79 | | 31,58 | |
| hsa-miR-1 | 40,00 | | 39,64 | | 38,56 | | 35,59 | |
| hsa-miR-299-3p | 38,92 | | 38,11 | | 40,00 | | 40,00 | |
| hsa-miR-142-3p | 33,84 | | 34,51 | | 34,28 | | 33,53 | |
| hsa-miR-338-3p | 27,74 | | 27,99 | | 27,81 | | 27,33 | |
| hsa-miR-584 | 33,68 | | 36,51 | | 40,00 | | 39,62 | |
| hsa-miR-377 | 40,00 | | 40,00 | | 40,00 | | 40,00 | |
| hsa-miR-216a | 40,00 | | 34,59 | | 36,59 | | 37,07 | |
| hsa-miR-206 | 40,00 | | 40,00 | | 40,00 | | 40,00 | |
| hsa-miR-921 | 40,00 | | 40,00 | | 40,00 | | 40,00 | |
| hsa-miR-513a-5p | 40,00 | | 40,00 | | 40,00 | | 40,00 | |
| hsa-miR-140-3p | 28,79 | | 28,69 | | 28,53 | | 28,11 | |
| hsa-miR-181a | 30,02 | | 29,63 | | 29,81 | | 29,74 | |
| hsa-miR-122 | 38,59 | | 39,05 | | 37,01 | | 33,53 | |
| hsa-miR-106a | 23,43 | | 23,15 | | 23,10 | | 23,27 | |
| hsa-miR-182 | 29,18 | | 28,19 | | 29,03 | | 27,92 | |
| hsa-miR-370 | 40,00 | | 40,00 | | 40,00 | | 38,51 | |
| hsa-let-7d-3p | 33,41 | | 34,22 | | 33,89 | | 32,91 | |
| hsa-miR-425-3p | 29,89 | 30,91 | | 30,89 | | 30,08 | |  |
| hsa-miR-450a | 40,00 | | 40,00 | | 40,00 | | 40,00 | |
| hsa-miR-411 | 40,00 | | 40,00 | | 35,17 | | 40,00 | |
| hsa-miR-216b | 38,78 | | 39,09 | | 35,33 | | 36,79 | |
| hsa-miR-106b | 29,04 | | 29,46 | | 29,60 | | 28,74 | |
| hsa-miR-886-3p | 40,00 | | 40,00 | | 40,00 | | 40,00 | |
| hsa-miR-510 | 34,56 | | 34,43 | | 36,20 | | 39,11 | |
| hsa-miR-212 | 34,44 | | 35,55 | | 40,00 | | 36,42 | |
| hsa-miR-525-5p | 40,00 | | 38,45 | | 40,00 | | 40,00 | |
| hsa-miR-589 | 34,51 | | 32,55 | | 34,79 | | 35,46 | |
| hsa-miR-576-3p | 34,71 | | 35,74 | | 35,66 | | 34,28 | |
| hsa-miR-583 | 40,00 | | 40,00 | | 40,00 | | 40,00 | |
| hsa-miR-483-3p | 25,69 | | 25,55 | | 25,18 | | 25,83 | |
| hsa-miR-582-5p | 29,35 | | 28,70 | | 29,71 | | 30,43 | |
| hsa-miR-886-5p | 40,00 | | 40,00 | | 40,00 | | 40,00 | |
| hsa-miR-33b | 34,18 | | 34,52 | | 36,61 | | 34,44 | |
| hsa-miR-193a-3p | 36,32 | | 37,37 | | 37,45 | | 36,31 | |
| hsa-miR-153 | 34,44 | | 35,11 | | 37,03 | | 34,71 | |
| hsa-miR-409-3p | 40,00 | | 40,00 | | 40,00 | | 40,00 | |
| hsa-miR-22 | 28,34 | | 27,83 | | 28,42 | | 28,35 | |
| hsa-miR-629 | 40,00 | | 31,98 | | 34,21 | | 32,54 | |
| hsa-miR-365 | 27,45 | | 27,43 | | 26,81 | | 26,38 | |
| hsa-miR-429 | 34,07 | | 31,91 | | 34,30 | | 32,38 | |
| hsa-miR-98 | 33,80 | | 32,99 | | 33,02 | | 32,22 | |
| hsa-miR-518a-3p | 40,00 | | 40,00 | | 40,00 | | 40,00 | |
| hsa-miR-137 | 40,00 | | 40,00 | | 35,88 | | 40,00 | |
| hsa-miR-508-3p | 40,00 | | 40,00 | | 40,00 | | 37,81 | |
| hsa-miR-539 | 40,00 | | 40,00 | | 40,00 | | 40,00 | |
| hsa-miR-148a | 27,84 | | 28,00 | | 29,26 | | 27,86 | |
| hsa-miR-146a | 26,71 | | 26,76 | | 27,57 | | 27,27 | |
| hsa-miR-139-5p | 34,59 | | 34,37 | | 36,09 | | 37,26 | |
| hsa-miR-373-5p | 40,00 | | 38,71 | | 34,74 | | 38,35 | |
| hsa-miR-149 | 30,99 | | 31,16 | | 31,02 | | 31,63 | |
| hsa-miR-642a | 40,00 | | 35,50 | | 40,00 | | 35,51 | |
| hsa-miR-31 | 34,40 | | 33,81 | | 35,53 | | 34,03 | |
| hsa-miR-451 | 40,00 | | 40,00 | | 40,00 | | 40,00 | |
| hsa-miR-100 | 40,00 | | 40,00 | | 40,00 | | 40,00 | |
| hsa-miR-27b | 28,25 | | 27,34 | | 29,39 | | 27,48 | |
| hsa-miR-523 | 40,00 | | 40,00 | | 40,00 | | 40,00 | |

**Table S2:** Target gene analysis for miR-24-3p and miR-151a-5p**.** Targets are predicted by TargetScan, miRanda and picTar with a CLIP-Seq overlap**.**

| **microRNA** | **geneName** | **position** |
| --- | --- | --- |
| hsa-miR-24-3p | AGPAT9 | chr4:84526851-84526857[+] |
| hsa-miR-24-3p | APPL2 | chr12:105567335-105567342[-] |
| hsa-miR-24-3p | ARID2 | chr12:46299414-46299420[+] |
| hsa-miR-24-3p | ARID5B | chr10:63856536-63856543[+] |
| hsa-miR-24-3p | ATAD2B | chr2:23974853-23974860[-] |
| hsa-miR-24-3p | ATG4A | chrX:107397074-107397081[+] |
| hsa-miR-24-3p | ATP6V0A2 | chr12:124243584-124243590[+] |
| hsa-miR-24-3p | AVL9 | chr7:32623748-32623754[+] |
| hsa-miR-24-3p | B3GNT1 | chr11:66113311-66113317[-] |
| hsa-miR-24-3p | BCL2L11 | chr2:111922250-111922257[+] |
|  | BCL2L11 | chr2:111924486-111924493[+] |
| hsa-miR-24-3p | BCL2L2 | chr14:23779522-23779529[+] |
| hsa-miR-24-3p | C10orf140 | chr10:21803646-21803652[-] |
| hsa-miR-24-3p | C22orf13 | chr22:24936436-24936442[-] |
| hsa-miR-24-3p | CCDC58 | chr3:122078601-122078608[-] |
| hsa-miR-24-3p | CCNI | chr4:77969251-77969257[-] |
| hsa-miR-24-3p | CCT3 | chr1:156278930-156278937[-] |
| hsa-miR-24-3p | CDKN1B | chr12:12875214-12875221[+] |
| hsa-miR-24-3p | CDV3 | chr3:133307200-133307206[+] |
|  | CDV3 | chr3:133308668-133308675[+] |
| hsa-miR-24-3p | CHIC1 | chrX:72904715-72904722[+] |
| hsa-miR-24-3p | CITED4 | chr1:41326927-41326934[-] |
| hsa-miR-24-3p | CLCN3 | chr4:170641562-170641568[+] |
| hsa-miR-24-3p | CNOT6 | chr5:180001376-180001382[+] |
| hsa-miR-24-3p | DCBLD2 | chr3:98515969-98515975[-] |
| hsa-miR-24-3p | DLGAP4 | chr20:35155544-35155551[+] |
| hsa-miR-24-3p | DLL1 | chr6:170591633-170591639[-] |
| hsa-miR-24-3p | DNAJB12 | chr10:74092694-74092701[-] |
|  | DNAJB12 | chr10:74094332-74094339[-] |
| hsa-miR-24-3p | ENTPD6 | chr20:25207017-25207023[+] |
| hsa-miR-24-3p | EXOC5 | chr14:57670455-57670462[-] |
| hsa-miR-24-3p | FAM175B | chr10:126523989-126523995[+] |
| hsa-miR-24-3p | FZD5 | chr2:208627840-208627847[-] |
| hsa-miR-24-3p | GBA2 | chr9:35737074-35737081[-] |
| hsa-miR-24-3p | H2AFX | chr11:118964696-118964702[-] |
|  | H2AFX | chr11:118965579-118965585[-] |
| hsa-miR-24-3p | HNF1B | chr17:36046978-36046985[-] |
| hsa-miR-24-3p | IGF2BP1 | chr17:47128657-47128663[+] |
| hsa-miR-24-3p | IL1R1 | chr2:102796135-102796141[+] |
| hsa-miR-24-3p | INSIG1 | chr7:155101029-155101035[+] |
| hsa-miR-24-3p | KCTD21 | chr11:77882418-77882425[-] |
| hsa-miR-24-3p | KDM5C | chrX:53222136-53222142[-] |
| hsa-miR-24-3p | KIAA0317 | chr14:75128961-75128967[-] |
| hsa-miR-24-3p | KIAA2018 | chr3:113368392-113368399[-] |
| hsa-miR-24-3p | KLF6 | chr10:3818970-3818976[-] |
| hsa-miR-24-3p | KLHL15 | chrX:24002996-24003002[-] |
| hsa-miR-24-3p | KPNA4 | chr3:160218451-160218457[-] |
| hsa-miR-24-3p | LIMD2 | chr17:61775481-61775488[-] |
| hsa-miR-24-3p | LMBR1L | chr12:49491297-49491304[-] |
| hsa-miR-24-3p | LYPLA2 | chr1:24121295-24121301[+] |
| hsa-miR-24-3p | MAGI1 | chr3:65340731-65340738[-] |
| hsa-miR-24-3p | MARCKSL1 | chr1:32799826-32799833[-] |
| hsa-miR-24-3p | MBOAT1 | chr6:20101553-20101560[-] |
| hsa-miR-24-3p | MIDN | chr19:1258160-1258167[+] |
| hsa-miR-24-3p | MNT | chr17:2290038-2290045[-] |
| hsa-miR-24-3p | MXI1 | chr10:112045616-112045623[+] |
| hsa-miR-24-3p | NEK4 | chr3:52745530-52745536[-] |
| hsa-miR-24-3p | NET1 | chr10:5499755-5499761[+] |
| hsa-miR-24-3p | NFXL1 | chr4:47849829-47849836[-] |
| hsa-miR-24-3p | OGT | chrX:70793677-70793683[+] |
| hsa-miR-24-3p | PER2 | chr2:239152890-239152896[-] |
| hsa-miR-24-3p | PHF23 | chr17:7138889-7138895[-] |
| hsa-miR-24-3p | PLOD2 | chr3:145788313-145788319[-] |
| hsa-miR-24-3p | POGZ | chr1:151377073-151377080[-] |
| hsa-miR-24-3p | PRDM1 | chr6:106556928-106556934[+] |
| hsa-miR-24-3p | PURA | chr5:139495945-139495952[+] |
| hsa-miR-24-3p | RAB5B | chr12:56386096-56386102[+] |
| hsa-miR-24-3p | RAB5C | chr17:40277160-40277166[-] |
| hsa-miR-24-3p | RALA | chr7:39747682-39747688[+] |
| hsa-miR-24-3p | RAP1B | chr12:69050989-69050996[+] |
| hsa-miR-24-3p | RAP2C | chrX:131339545-131339551[-] |
| hsa-miR-24-3p | RASA1 | chr5:86686938-86686944[+] |
| hsa-miR-24-3p | RBM27 | chr5:145665671-145665677[+] |
| hsa-miR-24-3p | RNF11 | chr1:51737056-51737062[+] |
| hsa-miR-24-3p | RNF138 | chr18:29709559-29709566[+] |
| hsa-miR-24-3p | RNF165 | chr18:44036681-44036688[+] |
| hsa-miR-24-3p | RNF2 | chr1:185069549-185069556[+] |
| hsa-miR-24-3p | S1PR1 | chr1:101705953-101705960[+] |
| hsa-miR-24-3p | SCML2 | chrX:18258177-18258184[-] |
| hsa-miR-24-3p | SESN1 | chr6:109308259-109308265[-] |
| hsa-miR-24-3p | SLC19A2 | chr1:169434064-169434070[-] |
| hsa-miR-24-3p | SMAGP | chr12:51639212-51639218[-] |
| hsa-miR-24-3p | SNN | chr16:11770457-11770463[+] |
|  | SNN | chr16:11772793-11772800[+] |
| hsa-miR-24-3p | SP1 | chr12:53805566-53805572[+] |
| hsa-miR-24-3p | SSH2 | chr17:27957850-27957856[-] |
| hsa-miR-24-3p | STC2 | chr5:172744219-172744226[-] |
| hsa-miR-24-3p | SUV420H2 | chr19:55859008-55859015[+] |
| hsa-miR-24-3p | TAOK1 | chr17:27871256-27871263[+] |
| hsa-miR-24-3p | TCERG1 | chr5:145890250-145890256[+] |
| hsa-miR-24-3p | TMEM161B | chr5:87491612-87491619[-] |
| hsa-miR-24-3p | TMEM194A | chr12:57453227-57453233[-] |
| hsa-miR-24-3p | TMEM9 | chr1:201104084-201104091[-] |
| hsa-miR-24-3p | TOP1 | chr20:39752100-39752107[+] |
| hsa-miR-24-3p | YOD1 | chr1:207220553-207220559[-] |
| hsa-miR-24-3p | ZBTB44 | chr11:130096900-130096906[-] |
| hsa-miR-24-3p | ZNF217 | chr20:52184214-52184221[-] |
| hsa-miR-24-3p | ZNF654 | chr3:88193712-88193719[+] |
| hsa-miR-24-3p | ZNF697 | chr1:120164770-120164777[-] |
| hsa-miR-24-3p | ZXDA | chrX:57934243-57934249[-] |
| hsa-miR-24-3p | ZXDB | chrX:57621099-57621105[+] |
| hsa-miR-425-5p | AFF4 | chr5:132211165-132211171[-] |
| hsa-miR-425-5p | AP3M1 | chr10:75883522-75883528[-] |
| hsa-miR-425-5p | ATP5G3 | chr2:176042922-176042928[-] |
| hsa-miR-425-5p | CBX6 | chr22:39260338-39260344[-] |
| hsa-miR-425-5p | CFL2 | chr14:35181501-35181507[-] |
| hsa-miR-425-5p | CPEB2 | chr4:15071041-15071047[+] |
| hsa-miR-425-5p | CREBZF | chr11:85372821-85372828[-] |
| hsa-miR-425-5p | DNAJB6 | chr7:157178490-157178496[+] |
| hsa-miR-425-5p | DYRK1A | chr21:38885850-38885857[+] |
| hsa-miR-425-5p | EFNB2 | chr13:107142410-107142416[-] |
| hsa-miR-425-5p | FOXJ3 | chr1:42642287-42642293[-] |
| hsa-miR-425-5p | GIT1 | chr17:27900528-27900534[-] |
| hsa-miR-425-5p | HDLBP | chr2:242168668-242168674[-] |
| hsa-miR-425-5p | HNRNPD | chr4:83275031-83275037[-] |
| hsa-miR-425-5p | LARP4B | chr10:855550-855556[-] |
| hsa-miR-425-5p | NRARP | chr9:140194966-140194972[-] |
| hsa-miR-425-5p | PDCD10 | chr3:167401831-167401837[-] |
| hsa-miR-425-5p | RNF145 | chr5:158584663-158584670[-] |
| hsa-miR-425-5p | SAMD4A | chr14:55259775-55259781[+] |
| hsa-miR-425-5p | SCAMP1 | chr5:77774075-77774082[+] |
| hsa-miR-425-5p | SLC39A10 | chr2:196602356-196602362[+] |
| hsa-miR-425-5p | SMEK1 | chr14:91924462-91924469[-] |
| hsa-miR-425-5p | SYNCRIP | chr6:86320980-86320986[-] |
| hsa-miR-425-5p | THRB | chr3:24160922-24160929[-] |
| hsa-miR-425-5p | WTAP | chr6:160176669-160176675[+] |
| hsa-miR-425-5p | ZBTB34 | chr9:129647836-129647842[+] |

**Table S3:** Target gene analysis for miR-425-5p. No CLiP-Seq data is available for this microRNA. Targets are therefore only predicted using TargetScan and only targets with evolutionary conserved target sites are listed.

| **microRNA** | **Target gene** | **Representative transcript** |
| --- | --- | --- |
| hsa-miR-151a-5p | WNT1 | NM_005430 |
| hsa-miR-151a-5p | SEZ6L | NM_001184773 |
| hsa-miR-151a-5p | NTRK2 | NM_001018065 |
| hsa-miR-151a-5p | N4BP1 | NM_153029 |
| hsa-miR-151a-5p | PHF15 | NM_015288 |
| hsa-miR-151a-5p | CASZ1 | NM_001079843 |
| hsa-miR-151a-5p | WIPI2 | NM_001033518 |
| hsa-miR-151a-5p | SOX12 | NM_006943 |
| hsa-miR-151a-5p | RIMBP2 | NM_015347 |
| hsa-miR-151a-5p | SLC12A5 | NM_001134771 |

1. If not analysed (N/A), the result was replaced with a threshold (C_T_) value of 40. [↑](#footnote-ref-1)
